# High Tibial Osteotomy: Review of Techniques and Biomechanics

**DOI:** 10.1155/2019/8363128

**Published:** 2019-05-02

**Authors:** Xiaoyu Liu, Zhenxian Chen, Yongchang Gao, Jing zhang, Zhongmin Jin

**Affiliations:** ^1^State Key Laboratory for Manufacturing System Engineering, School of Mechanical Engineering, Xi'an Jiaotong University, 710054 Xi'an, Shaanxi, China; ^2^Key Laboratory of Road Construction Technology and Equipment of MOE, Chang'an University, 710064 Xi'an, Shaanxi, China; ^3^Institute of Medical and Biological Engineering, School of Mechanical Engineering, University of Leeds, Leeds LS2 9JT, UK; ^4^Tribology Research Institute, School of Mechanical Engineering, Southwest Jiaotong University, 610031 Chengdu, Sichuan, China

## Abstract

High tibial osteotomy becomes increasingly important in the treatment of cartilage damage or osteoarthritis of the medial compartment with concurrent varus deformity. HTO produces a postoperative valgus limb alignment with shifting the load-bearing axis of the lower limb laterally. However, maximizing procedural success and postoperative knee function still possess many difficulties. The key to improve the postoperative satisfaction and long-term survival is the understanding of the vital biomechanics of HTO in essence. This review article discussed the alignment principles, surgical technique, and fixation plate of HTO as well as the postoperative gait, musculoskeletal dynamics, and contact mechanics of the knee joint. We aimed to highlight the recent findings and progresses on the biomechanics of HTO. The biomechanical studies on HTO are still insufficient in the areas of gait analysis, joint kinematics, and joint contact mechanics. Combining musculoskeletal dynamics modelling and finite element analysis will help comprehensively understand in vivo patient-specific biomechanics after HTO.

## 1. High Tibial Osteotomy

Knee joint is a very complex and important joint for load and motion, including the tibiofemoral (TF) joint and the patellofemoral (PF) joint. The stability of the knee joint is mainly dependent on the interaction by ligamentous and cartilaginous structures, meniscus as well as several muscles and tendons. Loads imposed on the tibiofemoral joint are over a few times the body weight (BW) during daily activities. And average peak resultant forces were highest during stair descending (346% BW), followed by stair ascending (316% BW) and level walking (261% BW) [1]. The medial-lateral force distribution is dependent on the tibiofemoral alignment and varies within different weight-bearing tasks. During a walking gait, the medial-lateral load distribution is changing on the tibia [2, 3]. And approximately 75% of the joint load passes through the medial tibial plateau during single-leg stance [4].

Osteoarthritis (OA) is nowadays the most common disease of joints in adults around the world [5]. OA is characterized by a progressive loss of articular cartilage accompanied by new bone formation and, often, synovial proliferation that may culminate in pain, loss of joint function, and disability [6]. Symptomatic OA is characterized by radiographic evidence along with persistent joint pain or stiffness [7]. Most common pattern of symptomatic OA within the knee is articular cartilage degeneration predominantly in the medial compartment [8, 9]. The joint degeneration further results in a varus deformity with increasing load transmission through the already degenerate compartment [4]. Furthermore, knee joint loading and kinematics have been found to be altered in patients with early knee OA during gait [10].

**Figure 1:**
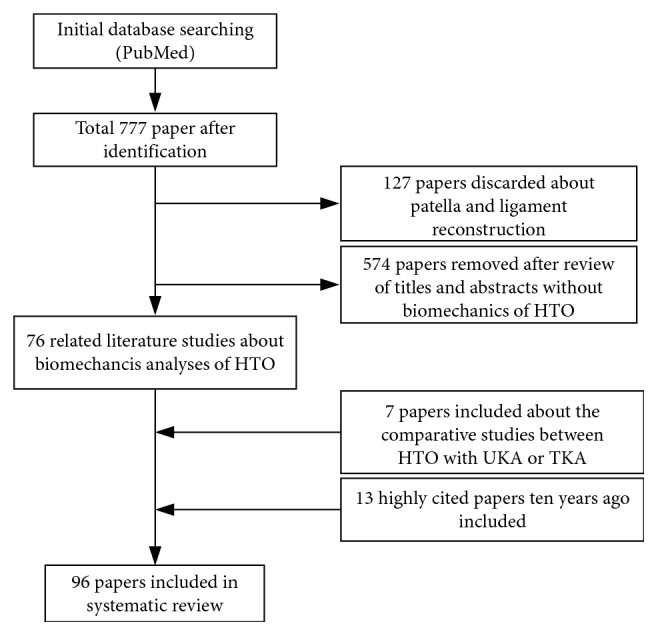
Flow chart.

A literature search was conducted using electric databases including the PubMed for English-language studies with full text from January 2008 to December 2018. When the search parameter used for PubMed database was “high tibial osteotomy” with full text for humans, 777 papers were identified. Most of them were to examine the functional clinical outcome after operations and analyze the determinants. What we need is biomechanics analyses of HTO. Hence, we excluded them, and we removed those studies about patella and ligament reconstruction. After screening, there were 76 related literature studies. Then seven papers about the comparative studies between HTO and UKA or TKA were included. So did the 13 highly cited papers ten years ago.  Figure 1 illustrates the flow chart of papers to explain the inclusion/exclusion criteria of studies.

Many conservative treatments of knee OA have been reported, such as medical treatment, foot orthoses, knee braces, and muscle strengthening. Those treatments can prevent or slow the progression of medial knee OA [11]. However, no conclusive evidences have been confirmed in many previous studies on the effectiveness of any braces and orthoses for patients with medial knee osteoarthritis [12]. And the optimal choice for an orthosis remains unclear, and long-term implications are lacking [13]. On the contrary, total knee arthroplasty (TKA) has been established as a very successful and commonly performed procedure for primary and secondary osteoarthritis. However, compared to medial unicompartmental knee arthroplasty (UKA) and high tibial osteotomy (HTO), active and demanding activities seem more unlikely after TKA [14, 15]. HTO showed an improved indication for active patients with a good range of motion of the knee [14, 16]. Although there are no significant differences in the clinical outcome between UKA and HTO at 12 months and 2-year follow-up, the advantages of HTO is the preservation of the knee joint as long as possible, a large corrective effect of the mechanical axis, and the avoidance or postponement of knee replacement [17–19]. The main indications for HTO and UKA are summarized and listed in Table 1 [20]. Most HTO patients returned to sport and work after knee osteotomy. From 10 to 22 weeks, almost all patients returned to the same or a higher workload [21].

Selection of the ideal patient is an important factor in achieving good results with HTO. Based on the previous findings, the ideal candidate for an HTO is a young patient (<60 years of age), with no severe articular destruction (Ahlback grade III or more according to the Ahlback classification), isolated medial osteoarthritis, and good range of motion and without ligamentous instability [22]. The contraindication is ≥15° of flexion contracture, joint instability together with ≥1 cm lateral tibial thrust, ≥20° of correction, rheumatoid arthritis, and advanced patellofemoral arthritis [23]. With the improvements in soft-tissue preparation, advances in surgical techniques, neither the patients' weight and age nor the genesis of deformity has been found to influence the rate of complication from a large number of postoperative databases [24–26].

Although HTO has recently become advocated and used to treat osteoarthritis around the knee, it still causes some complications such as nonunion, tibial plateaus fracture, lateral cartilage degeneration, plate breakage, and so on. Stiffness is uncommon if preoperative motion is satisfactory [27]. Furthermore, a small percentage of patients treated with HTO (4% to 26%) do not have satisfactory pain relief, and this is the primary reason for revision to TKA [28–30]. Ultimately, majority of complications and dissatisfaction are closely related to the biomechanics of HTO. The key to improve the postoperative satisfaction and long-term survival is the understanding of the vital biomechanics of HTO.

## 2. Alignment Principle

**Figure 2:**
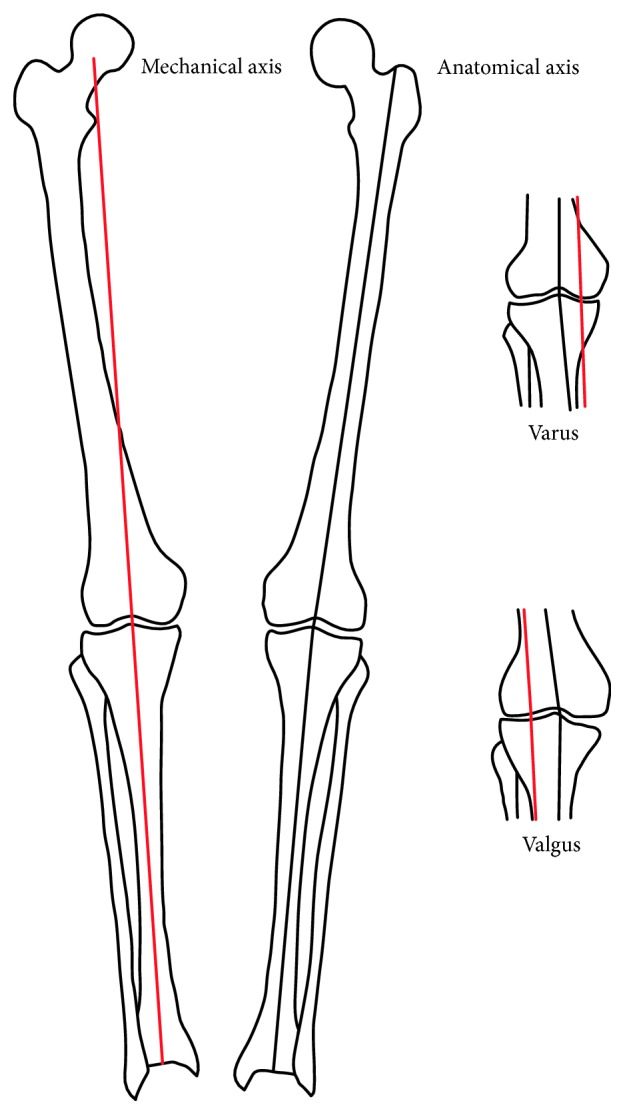
Radiographic lower limb alignment assessment. The mechanical axis of the limb (red line) is defined by a line from the center of the femoral head to the medial tibial spine and a line from the medial tibial spine to the center of the ankle. The weight-bearing line (also represented by the red line, as this knee has normal alignment of 0°) is defined by a line from the center of the femoral head to the center of the ankle joint. The anatomic axis of the limb (black line) is defined by mid-diaphyseal lines in the femur and tibia. In a varus knee, the weight-bearing axis passes medial to the medial tibial spine. In a neutral knee, the weight-bearing axis passes through the medial tibial spine. In a valgus knee, the weight-bearing axis passes lateral to the medial tibial spine [32].

The ideal mechanical axis passes from the center of the hip, through the knee, to the center of the tibiotalar joint [31]. The orientation of the normal anatomic axis of the knee is 5° to 7° valgus [32]. In addition, the articular surface of the tibia averages 3° varus and that of the femur 2° to 3° of valgus relative to the mechanical axis [33]. Schematic limb alignment assessment is shown in Figure 2. In neutral alignment, the knee moment in the coronal plane causes approximately 55 ∼ 70% of knee load to be transmitted on the medial compartment during the stance phase of gait [34]. With varus alignment, this imbalance is exacerbated so that a deviation of 1° varus from the neutral alignment increased the medial load by 5% [35]. Analyses of interindividual variations revealed a linear correlation with limb alignment [35]. In a longitudinal observational study, the varus alignment of more than 2° considerably increased the probability of developing OA in a rather short period of time [36].

The biomechanical objective of HTO is to realign the weight-bearing line (WBL) in the coronal plane. The aim is to achieve the shift of the weight-bearing line from the arthritic compartment to the opposite tibiofemoral healthy compartment [37]. Overall, leg alignment is a crucial factor for the force distribution in the knee joint [38]. The decrease of load in the diseased compartment of the tibial plateau reduces knee joint pain and delays progression of osteoarthritis [39, 40].

The reported success rates of HTO are inconsistent with the long-term survivals and satisfaction of this procedure. Although a consensus is that ideal opening wedge HTO produces a decompression of the medial joint compartment, optimal amount of alignment correction of the lower limb remains unknown, which may result in the discrepancies among the surgeries [10]. Fujisawa et al. [36, 41] recommended to align the WBL of HTO through the 65%–70% coordinate of the width of the tibial plateau, which has been refined recently to 62.5% (range 62% ∼ 66%). An average overcorrection of 3° valgus was supported by previous studies long ago. However, excessive overcorrection would lead to worse functional outcomes and degeneration in the lateral compartment, while undercorrection could not relieve the pain of the medial compartment [36, 42, 43].

The accurate correct angle is dependent on the patient's physical condition and the severity of arthritis generally. The reason why overcorrected knees are applied widely is that patients with a valgus angle of 3° and more had the best outcome and highest postoperative survival rate [37, 44]. However, this recommendation is only based on one noncomparative retrospective study, and the recommendations based on higher evidence levels do not exist. There is no reasonable way to evaluate the optimal angle of osteotomy before operation, which is the most important for limb alignment and long-term results. Furthermore, there are no significant differences in terms of the ratio of cartilage repair in the medial compartment of the tibiofemoral joint between 17 overcorrected knees with mean tibiofemoral angle of 165° ± 1° and 54 moderately corrected knees with mean tibiofemoral angle of 170° ± 2° after open-wedge HTO [45].

Compared to the two-dimensional (2D) alignment in the coronal plane, the three-dimensional (3D) alignment is a potential method to achieve better results in short and long terms after HTO surgery [46]. The 3D alignment method shows better correction on the knee load bearing, and the most important factor in HTO is observing the WBL in a 3D environment. The posterior tibial slope angle may be increased without the consideration of the change in the sagittal plane. The 3D alignment method can effectively avoid such postoperative complication, and it is worthy of further study and clinical verification. 3D printing technique has been introduced recently in HTO, and good radiological results have also been obtained [47].

Specogna et al. [48] reported the effects of the dynamic measurement on the tibiofemoral angle (TFA) during the gait cycle, which is different from the static alignment. Furthermore, standing full-length alignment (SFLA) and supine radiographs alignment were compared. The measured TFA by single-limb standing radiographs was significantly greater (−8.7° ± 4.0°) than that by double-limb standing radiographs (−7.1° ± 3.8°) and by supine radiographs (−5.5° ± 2.8°) [49]. Hence, the standing alignment may be better than the supine radiographs alignment, and the dynamic alignment may be superior to static measures.

## 3. Open-Wedge or Closed-Wedge HTO

**Figure 3:**
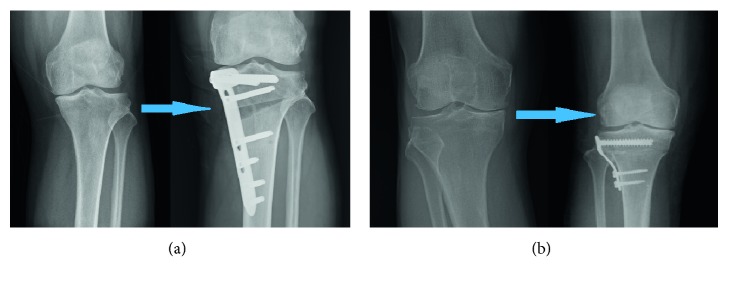
Schematic of open-wedge HTO (a) and closed-wedge HTO (b) of a knee with varus deformity.

Open-wedge (OW) and closed-wedge (CW) HTO are different osteotomy techniques (Figure 3). The advantages and disadvantages in clinical results between OWHTO and CWHTO are compared in Table 2. Recent studies have shown that OWHTO has several advantages over CWHTO, including higher accuracy of correction, better survival at ten years, wider range of motion, less soft-tissue dissection, and more reserve of the proximal tibiofibular joint [19, 53, 54, 56]. However, OWHTO also increases the posterior slope angle and limb length and decreases the patellar height [19, 51, 54, 55]. Besides, autologous iliac bone graft is unnecessary for patients in whom the opening wedge is <12.5 mm [50, 57]. CWHTO trend led to a higher incidence of opposite cortical fracture [19].

Prodromos and Andriacchi [58] found that patients with a low knee adductor moment had better clinical results according to gait analysis after HTO. Deie et al. [52] reported that OWHTO reduced knee varus moment and lateral thrust, whereas CWHTO had little effect on reducing lateral thrust. According to their results, opening the depressed medial proximal tibia is thought be a more reasonable procedure in terms of correcting the deformed lesion than closing the intact lesion of the proximal tibia from a biomechanical aspect. Hence, medial OWHTO has been an effective and appealing surgical procedure intended to treat medial compartment osteoarthritis in young and active patients with proximal varus knees [59].

## 4. Fixation Plate

HTO results in a highly unstable structure of the proximal tibia, which is the potential source of mechanical failure of plates and screws. Consequently, use of the fixation devices and optimal designs are essential to the success of HTO, especially for overweight or full weight-bearing patients.

Majority of studies have investigated the fixation plate design of HTO. Currently, the commercial implants for the treatment of medial knee joint osteoarthritis are TomoFix small stature, TomoFix standard, Contour Lock, iBalance, and second-generation PEEKPower [60]. The use of locking screws can stabilize the construct and decrease the implant and bone stresses [61]. The one-leg system with locking screws can be used for the majority of the patients without heavy bodyweight and poor bone quality. For the shape design, a two-leg system is suggested for the patients with heavy load demands and greater proximal tibial size. T- and I-shaped plates can provide a wider base for supporting the HTO wedge even without the use of locking screws, thus significantly enhancing construct stiffness and suppressing wedge fracture [62]. A more concave tibial profile and/or reduced distraction angle necessitates a higher compressive load to elastically deform the plate, thereby deteriorating the lateral-hinge fracture risk [63]. A precontoured plate is recommended by surgeons when the proximal tibia is highly concave, and the distraction angle is insufficient to stretch the tibial profile. Diffo Kaze et al. [60] reported a novel anatomically contoured implant called “Activmotion” which can provide a better mechanical stability and strength. Ideal implants with a metaphyseal slope should adapt to the tibia anatomy and position more anteriorly on the medial compartment of the proximal tibia. Furthermore, the position on the proximal tibia of the fixation plate is also important. More comparable performance was found when TomoFix plate was placed more medially than the T- and I-shaped plates [62]. Therefore, if a single plate and a smaller incision are considered, the medial implant position of the TomoFix plate is appropriate as a better alternative for stabilizing the medial HTO wedge [62]. In addition, the difference of having a drill hole or not at the end of a horizontal osteotomy was investigated, and the effect on reducing the risk of lateral cortex fracture was not significant, especially for older patients [64]. A cadaveric experiment produces similar conclusions that there was no significant difference in the strains on the lateral cortex during OWHTO between the pilot hole and no-hole conditions [65].

The present findings about the biomechanics of the fixation plate showed that implant position and the geometry are vital parameters to maintain stability. The current plate design should be modified to the surface geometry of the postcorrection for the proper fitting [66]. As the correction degree increases, the plate should be bent at both ends of the opening gap in the coronal plane [67]. Patient-specific design of the fixation plate of HTO may be an alternation in future.

## 5. Kinematics

Medial compartment OA with varus deformity leads to the changes in kinematics of gait and joint movement. In addition to restoring the normal alignment of the lower limb, HTO is also successful in modifying the osteoarthritic gait [68]. However, there are some discrepancies in analyses about subjects, methodologies, and outcomes. Furthermore, the changes in gait could have diverse effects on the trunk, nonoperated limb, and hip and ankle joint in the operated limb after HTO [69]. Recent kinematical studies in gait are summarized in Table 3. HTO does not alter the time-distance parameters of gait at one year postoperatively; however, patients have improved perception of their walking ability [10]. Walking speed and stride length were increased after HTO [68, 70]. The range of motion of the knee joint was increased and maintained for 5 years after HTO with anterior cruciate ligament (ACL) reconstruction [71]. The corrected approximately neutral alignment in HTO produced substantial changes in dynamic loading and function of knee joint [73]. HTO presented positive results in joint kinematics after postoperative 6 months, not only in the coronal plane but also in the sagittal and axial planes [72]. Leitch et al. [70] found medial OWHTO resulted in decreased flexion and internal rotation during both level walking and stair ascent. In addition, gait modifications are an important approach to reduce the knee adduction moment (KAM) without necessarily decreasing the medial compartment force [74]. However, in general, few studies are performed to investigate the gait modification and joint movement after HTO.

## 6. Knee Joint Moment and Force

Balancing loads between medial and lateral compartments is an important factor in improving the long- or short-term survival rates of HTO. Ideally, an appropriate correction achieves a minimum overcorrection from baseline alignment necessary for adequate medial unloading, whilst avoiding overloading on the lateral compartment cartilage. The current research studies reported that KAM and knee flexion moment (KFM) after HTO of the surgical knee were decreased significantly [68, 70–72, 75, 76] and medial OWHTO resulted in a decrease in the KAM during both level walking and stair ascent [70]. However, in coronal, sagittal, and transversal planes, the change of the KAM is inconsistent in different reports [72]. Recent knee moment studies in gaits are shown in Table 4.

Although KAM was a surrogate for knee contact force (KCF), it well suited to predict the medial force ratio throughout the whole stance phase or medial force during the early stance phase [77]. However, KAM was not sufficient to predict joint loading at the end of the stance, where external KAM contributed substantially to the loading, especially in early OA [78, 79]. Some findings suggested that the KCF predicted by a novel musculoskeletal simulation routine provides a more helpful metric than the KAM [75]. Lerner et al. [80] found that each 1° of TF alignment deviation altered the first peak medial KCF by 51 N, whilst each 1 mm of medial-lateral translation of the compartment contact point position altered the first peak medial KCF by 41 N. KCF can be used to identify early knee OA development prior to the onset of radiographic evidences [81]. However, currently, the tolerance of the in vivo joint cartilage to stress and the relationship between joint loading and the osteoarthritis pain and disease progression remain in dispute, which induces a challenge for determining appropriate loading for any individual.

Some studies analyzed the biomechanical effects of varus knee deformity on the stress distribution in the articular cartilage. Martay et al. [82] supposed the contact stresses on the medial compartment were already “too high” for HTO patients, and it was necessary to decrease the medial contact stresses and maintain relatively lower lateral contact stresses to avoid damaging the lateral tissues. They proposed correcting the weight-bearing axis to 55% tibial width (1.7°–1.9° valgus) optimally distributes medial and lateral contact stresses [82]. Nakayama et al. [83] found a large amount of correction in OWHTO with a resultant joint-line obliquity of 5° or more may induce excessive shear stress to the articular cartilage. Zheng et al. [84] also found that balanced loading occurred at angles of 4.3° and 2.9° valgus for the femoral and tibial cartilage, respectively. The study of Trad et al. [85] suggested that a balanced stress distribution between two compartments was achieved under a valgus hypercorrection angle of 4.5. The main conclusions of current relative studies are shown in Table 5. However, there are quite a few research studies related to the influence of limb alignment on medial-lateral loading and the effect of axis correction angle on stress distribution on the tibial plateau after HTO. How much the contact stresses on medial compartment cartilage should be reduced to prevent progression is still unknown, while what a threshold is beyond resulting in increasing contact stresses on lateral compartment also is unclear.

Some researchers found that knee joint with exposed bone was concluded to be partially or entirely covered by newly regenerated cartilage after HTO [86, 87]. However, one study reported no significant differences in terms of the ratio of cartilage repair in the medial compartment of the tibiofemoral joint between the overcorrected knees and corrected knees after OWHTO [45]. It is unclear whether the “safety corrective range” or the golden standard for the OA exists. The effect of excessive stress on soft-tissue wear or repair and the remodeling process after corrective osteotomy is still unknown. There remains a lack of quantitative research about the change in knee contact mechanics of HTO.

## 7. Research Method

Gait analysis, musculoskeletal modelling, and finite element analysis (FEA) are the main research methods for investigating the biomechanics of HTO in the above-mentioned study. Joint kinematics can be mainly measured using two techniques. In vivo joint kinematics and gait pattern are most commonly determined using a marker-based 3D motion-capture system during walking, stair ascent, and squat activities [71, 88]. But the subjects with high BMI are not suitable for this technology because soft-tissue motion relative to bony landmarks can introduce errors [89]. Dual fluoroscopy is more accurate with excellent precision than marker-based 3D motion capture [90–92]. Dual fluoroscopy captures 3D joint kinematics by registering 3D surface reconstructions to the 2D images acquired using fluoroscopes. The primary disadvantages of this technique are the technical challenge and radiation exposure. In spite of existing limitations, both techniques provided an approach to investigate a subject's gait pattern and in vivo joint kinematics of HTO.

An array of available musculoskeletal modelling software, for example, Anybody [89] and OpenSim [93], has been used to obtain in vivo biomechanics of the human body. The kinematical data from gait analysis was the important input condition for inverse kinematic analysis and inverse dynamics analysis of the musculoskeletal multibody dynamics model. Musculoskeletal models could estimate subject's muscles forces, joint moments, and joint reaction forces as well as joint kinematics by solving the muscle redundancy problem. The biomechanics information of joint loading and motion were the vital boundary condition of FEA. The musculoskeletal modelling method would afford a wealth of understanding on the influence of gait patterns on muscles and joint force magnitudes, a strong platform of quantifying the biomechanics of HTO. Hu et al. [94] and Chen et al. [3] investigated the biomechanics of the natural knee joint and total knee replacement using subject-specific musculoskeletal multibody dynamics models. Therefore, the musculoskeletal modelling method is easily used to investigate the biomechanics of HTO under musculoskeletal dynamics environment.

FEA of joint contact mechanics has already provided insight into the mechanical causes of OA [7]. Subject-specific FEA of joint contact mechanics also provides noninvasive, patient-specific recommendations of HTO correction angle. But few studies have investigated the effect of HTO correction angle on the stress distribution of the articular cartilage in the knee joint [82–85]. Zheng et al. [84] introduced a platform for noninvasive, patient-specific preoperative planning of the osteotomy for medial knee osteoarthritis using CAD and FEA. Multiobjective optimization could be used to identify the final alignment that balanced medial-lateral compressive and shear forces. However, limitations of simple materials' parameters for intact cartilage or meniscus were adopted in all the aforementioned studies. Saarakkala. et al. [95] found that maximum principal stresses and strains within the articular cartilage of the knee joint during walking are highly sensitive to the material parameters of the cartilage. However, the biphasic mechanics of the articular cartilage was rarely considered in FEA of HTO. The study of Meng et al. [96] took into account the complex biphasic contact interactions of the cartilage and menisci to characterize the time-dependent contact behavior of the tibiofemoral joint under body weight. Furthermore, most FE models only applied static loading and omitted joint kinetics during motion. Because many orthopedic pathologies altered the joint motion and force, those changes should be incorporated into the FE model as accurately as possible [7]. In the future, with the advantages of gait analysis, musculoskeletal modelling, and FEA, the “safety corrective range” of HTO can be determined.

## 8. Conclusion

The patient's gait pattern after HTO is modified based on the limb alignment, which would further influence the knee adduction moment and medial-lateral contact forces and consequently the contact stresses of the cartilage on the medial-lateral compartments of the tibiofemoral joint. Biomechanical environment of HTO is crucial for understanding the complications of HTO, and improving surgical accuracy. However, biomechanical studies on HTO are still insufficient on gait analysis, joint kinematics, and joint contact mechanics. The biomechanical relationships between the alignment and plate breakage, cartilage degeneration, nonunion, and others are still unclear. The “safety corrective range” is still unknown. Integration of gait analysis, musculoskeletal dynamics modelling, and FEA will help comprehensively understand in vivo patient-specific biomechanics information of HTO.

## Figures and Tables

**Table 1:** Indications for UKA, HTO, and overlaps between treatments.

	UKA	HTO or UKA	HTO
Age	>55 years	55–65 years	<65 years
Activity level	Low demands	Moderately active	Active
Weight (BMI)	<30	Any	
Alignment	0–5°	5–10°	5–15°
AP instability	No to grade I	No to grade I	Any
ML instability	No to grade I	No to grade I	No to grade II
ROM	Arc 90° and <5° flexion contracture	Arc 100° and <5° flexion contracture	Arc 120° and <5° flexion contracture
Arthrosis severity	Any	Ahlback II	Ahlback I-II

UKA = medial unicompartmental knee arthroplasty; HTO = high tibial osteotomy; BMI = body mass index; AP instability = anteroposterior instability; ML instability = mediolateral instability; instability grading: according to the American Medical Association (grade I = 0–5 mm; grade II = 5–10 mm; grade III = >10 mm; no hard stop); arthrosis severity = medial compartment arthrosis according to Ahlback classification, assuming that lateral and patellofemoral compartments are intact.

**Table 2:** Comparison of the clinical results between open-wedge (OWHTO) and closed-wedge (CWHTO).

Year	Papers	Patients		OWHTO	CWHTO
		OW	CW		
2014	Duivenvoorden et al. [50]	45	47	More complications	More early conversions to total knee arthroplasty with six years
2014	Van Egmond [51]	25	25	Patella baja leads to patellofemoral complaints and worse results	Better satisfactory and score with an average of 7.9 years
2014	Deie et al. [52]	9	12	Reduced knee varus moment and lateral thrust	Little effect on reducing lateral thrust
2015	Duivenvoorden et al. [53]	112	354	Higher survival ratio, 15% serious adverse events, 13% adverse events	13% serious adverse events, 6% adverse events
2016	Sun et al. [19]	740	743	Increased the posterior slope angle and limb length; decreased the patellar height; higher accuracy of correction	Higher incidence of opposite cortical fracture
2017	Wu et al. [54]	1274	1308	Wider range of motion; greater posterior tibial slope angle; lesser patellar height	No significant difference in HKA and mean angle of correction
2018	Lee et al. [55]	127	175	The increase in leg length had a positive correlation with the degree of correction	The decrease in leg length was negligible

**Table 3:** Changes in kinematics after HTO.

Year	Author	Patients	Duration	Gait parameter
2013	Lind et al. [68]	11 male patients with medial OA	Before 12 months and after medial OWHTO	(1) Mean maximum varus angle during stance was reduced from 13.5° to 5.4°(normal 6.8°) **(**2) Walking speed increased significantly postoperatively. **(**3) Maximum knee flexion increased significantly **(**4) Stride length increased from 1.37 m preoperatively to 1.48 m postoperatively **(**5) The mean radiological mechanical alignment was changed (pre-op: 172°, post-op: 180°)

2015	Leitch et al. [70]	14 patients with varus alignment and OA	Before 6 and 12 months after OWHTO	(1) Speed increased after surgery. **(**2) The peak external rotation angle was increased after surgery

2015	Marriott et al. [71]	33 patients with varus	Before 2 and 5 years after ACL reconstruction and HTO	(1) The means of valgus, flexion, and internal rotation angle increased by 7.79°, 3.80°, and 7.07°, respectively, with 5 years **(**2) The means of extension and external rotation angle decreased by 2.14° and 5.88°, respectively, with 5 years

2017	Da Silva et al. [72]	21 patients with OWHTO compared to the control group (16)	Short-term results of HTO of 6 months	(1) No significant changes in stride length and speed were observed in the post-op period **(**2) The foot external rotation angle decreased significantly in the axial plane (25.5°–11.5°) **(**3) Knee varus angle significantly reduced in the coronal plane (pre-op: 11.6°; post-op: 4.3°)

2017	Morin et al. [10]	21 HTO patients	Preoperatively and at 1 year postoperatively	(1) The preoperative median of 7° varus (1–11°) was corrected to 3° valgus (0–6°) **(**2) Time-distance gait parameters, such as step width and walking speed, did not change 1 year after surgery **(**3) The patients' subjective perception of their walking ability improved

**Table 4:** Changes in knee moment after HTO.

Year	Author	Patients	Duration	Force or moment analysis	The influence of nonsurgical limb
2010	Bhatnagar and Jenkyn [76]	30 HTO patients	Pre-HTO, 6 and 12 months post-HTO	(1) ML and MLR were reduced significantly by 0.56% BW and 1.0, respectively **(**2) First peak of an EKAM during stance phase was reduced significantly by 1.70% BW *∗* ht **(**3) No significant difference was observed between 6 and 12 months post-HTO	—

2013	Meyer et al. [75]	A single subject: Implanted with a tibial prosthesis	—	(1) Total contact force may be changing **(**2) KAM is not a suitable indicator of medial contact force in situations	—

2013	Lind et al. [68]	11 male patients with medial OA	Before 12 months and after OWHTO	(1) Mean maximum KAM reduced from 3.9 to 2.7 (% Bw *∗* ht) **(**2) Maximum of EKFM increased significantly	KAM increased postoperatively from 3.3 to 4.1 (% Bw *∗* ht)

2015	Leitch et al. [70]	14 patients with varus and OA	Before, 6 and 12 months after OWHTO	The peak KAM, KFM, and IRM all decreased significantly after HTO during walking and stair ascent with sustained (12 months) changes in all three orthogonal planes	IRM was higher during stair ascent, while the peak KAM was lower

2015	Marriott et al. [71]	33 patients with varus	Before, 2 and 5 years after ACL reconstruction and HTO	(1) The EKAM and KFM in the surgical limb decreased significantly in the peak. **(**2) Substantial improvements were maintained at 5 years in all 3 planes during walking.	(1) KAM increase slightly. **(**2) KFM decreased.

2017	Da Silva et al. [72]	21 patients with OWHTO	Short-term results of HTO of 6 months	(1) The peak of KAM and KFM was reduced and close to the values of the control group in the coronal plane **(**2) The peak KFM and the KEM was increased significantly in the sagittal plane	—

**Table 5:** Knee contact mechanics of HTO.

Year	Author	Data	Conclusion	Limitation
2017	Nakayama et al. [83]	(1) The 3D bone model was derived from human bone digital anatomy media and only included the distal femur and proximal tibia	(1) The obliquity angle increases laterally directed shear stress **(**2) An obliquity angle of 5° or more increases shear stress in the medial compartment; the maximum shear stress value in the medial cartilage increased from 1.6 MPa for the normal knee to 3.3, 5.2, and 7.2 MPa in the joint-line obliquity models with 5°, 7.5°, and 10° of obliquity, respectively	(1) Due to the data source, these results cannot be generalized and applied to all patients with osteoarthritis undergoing osteotomy **(**2) The knee model used for the FEA omitted meniscus and unfirming the thickness of cartilage to avoid excessive complexity in calculation

2017	Zheng et al. [84]	(1) MRI data of a healthy participant. **(**2) Gait analysis and force-platforms data during ten walking trials	(1) Providing a platform for noninvasive, patient-specific preoperative planning of the osteotomy for medial compartment knee osteoarthritis **(**2) Balanced loading occurred at angles of 4.3° and 2.9° valgus for the femoral and tibial cartilage, respectively	(1) Did not consider the whole gait cycle **(**2) Did not apply muscle forces within their individual lines of action. **(**3) Simulation on a healthy knee with intact menisci.

2018	Trad et al. [85]	The 3D model of the right lower limb was extracted from a 3D anonymous human skeleton	(1) The model agreed with the experimental and numerical results **(**2) By changing the correction angle from 0 to 10 valgus, the von Mises and the shear stresses decreased in the medial compartment and increased in the lateral compartment **(**3) A balanced stress distribution between two compartments was achieved under a valgus hypercorrection angle of 4.5	(1) The use of the geometry of a knee model artificially created and not the one specifically developed for a pathological knee **(**2) Without studying the dynamic behavior **(**3) Neglecting the cancellous bone and the muscle forces **(**4) All the knee components were considered as linearly homogeneous isotropic material

2018	Martay et al. [82]	(1) MRI data of three healthy subjects. 2. Marker trajectory data and GRF data during level walking	Correcting the weight-bearing axis to 55% tibial width (1.7°–1.9° valgus) optimally distributes medial and lateral stresses/pressures	(1) Simulation on healthy knees **(**2) Using simple material behaviors **(**3) Validating their model creation method using porcine specimens **(**4) Without studying the dynamic behavior
